# Conceptual framework of coaches’ decision-making in conventional sports

**DOI:** 10.3389/fpsyg.2024.1498186

**Published:** 2025-01-09

**Authors:** Edvard Kolar, Roberto Biloslavo, Rado Pišot, Saša Veličković, Matej Tušak

**Affiliations:** ^1^Science and Research Centre Koper, Institute for Behavioral Economics, Koper, Slovenia; ^2^Science and Research Centre Koper, Institute for Kinesiology Research, Koper, Slovenia; ^3^Faculty of Sport and Physical Education, University of Niš, Niš, Serbia; ^4^Faculty of Sport, University of Ljubljana, Ljubljana, Slovenia

**Keywords:** sport training, coaches, decision-making behavior, types of decisions, conceptual framework

## Abstract

**Introduction:**

A coach’s managerial and pedagogical tasks in the sports training process constitute the substantive core of their work, while decision-making serves as the fundamental method underpinning these tasks. Some decisions made by coaches result from deliberate, analytical thinking, which involves extensive information gathering, analysis, and discussion. Others, however, are made quickly and spontaneously, triggered by unforeseen situations during training or competition that demand immediate action. Consequently, the purpose of this study is to develop a conceptual framework for understanding coaches’ decision-making behavior in conventional sports. This framework aims to establish appropriate relationships between the various decisions coaches make during the training process and theoretical concepts related to decision-making, both in general and within the coaching context.

**Methods:**

To design the research, we used the methodology of a conceptual paper and a “model paper” approach, which seeks to build a theoretical framework that predicts relationships between distinct research concepts and scientific disciplines, aiming to integrate them into a cohesive model of coaches’ decision-making behavior.

**Results:**

The proposed conceptual framework encompasses a comprehensive range of situations that may arise during the sports training process and potential ways to address them. This framework identifies different types of decisions and characteristics associated with coaches’ decision-making behavior. It incorporates various sport-specific and general theories of decision-making and cognitive functioning to offer a deeper understanding of how coaches process and execute decisions in diverse contexts.

**Discussion:**

The developed conceptual framework outlines three primary types of decisions—strategic, tactical, and operational—each playing a distinct role in the broader sports training process. These decisions are based on different cognitive processes, which manifest in varied decision-making behaviors and are reinforced by specific leadership styles. The practical value of this framework lies in its potential application for selecting appropriate experts to address the diverse decision-making scenarios encountered in sports training. This ensures the alignment of decision-making styles with the requirements of specific training situations, thereby enhancing the effectiveness and outcomes of the coaching process.

## 1 Introduction

The performance of an athlete in elite-level sports can only be measured by the results achieved in major international competitions, which heavily depend on the athlete’s proper preparation during the process of sports training. Sports training is defined as a long-term, transformational process that involves the athlete as a multidimensional system. An athlete’s performance is primarily determined by two key factors: (1) the development of their capabilities (dimensions), such as knowledge, skills, personal characteristics, and motivation, (2) and the successful and efficient management of the sports training process (Kolar et al., 2006, p. 11). The overarching purpose of the training process is to transform and adapt the athlete’s capabilities to meet the specific demands of their sport.

Despite the complexity, uncertainty, and increasing demands for excellence in sports training—which necessitate an interdisciplinary approach involving highly trained experts from diverse fields (e.g., physiology, biomechanics, medicine, nutrition, psychology) (Wilson and Kiely, 2023, p. 2)—the coach occupies the central role in managing the process. Nash and Collins (2006, p. 467) describe coaches as managers of the coaching process, technical advisors, tacticians, and educators. Coaches are responsible for the following: (1) managing the organizational process, which includes planning, organizing, implementing (pedagogical processes), controlling, and evaluating both the results and the training process. (2) Coordinating all involved experts and athletes, ensuring alignment and collaboration. (3) Delegating tasks and activities effectively to optimize the training process.

These three core managerial responsibilities represent the substantive content of a coach’s work. However, the fundamental method by which coaches carry out these tasks is decision-making (hereinafter DM) (Abraham et al., 2006, p. 549; Kolar and Tušak, 2022, p. 49; Wilson and Kiely, 2023, p. 2).

Various authors argue that coaching is fundamentally a DM process (Abraham and Collins, 2011, p. 367; Lyle and Muir, 2020, p. 1), while coaches’ DM has been identified as a key element of their practice (Kaya, 2014, p. 333; Coutts, 2017, p.717; Till et al., 2019, p. 14), described as the “hallmark” of an expert coach (Nash and Collins, 2006, p. 466), the “first among equals” among the essential skills a good coach must possess (Post and van Gelder, 2023, p. 25), and a defining characteristic of coaching expertise (Harvey et al., 2015, p. 152).

DM is a process resulting in a decision (Tomić, 2007, p. 188). Heller and Hindle (2001, p. 153) wrote that “decisions are an essential part of our lives, both in the work environment and outside of it, and that they are made by those who are responsible for choosing between two or more alternatives.” A decision, as a result of a DM process, can be defined as “a judgment or choice between two or more options that develops from an infinite number of situations, from solving a problem to taking action in a certain direction” (Heller and Hindle, 2001, p. 154). The DM process is deeply affected by the interplay between two cognitive systems, namely System 1 and System 2, as delineated by Kahneman (2017, p. 576). System 1 (also System-X, intuitive or heuristic system), which operates quickly and automatically with little to no effort and no sense of voluntary control, can have an outsized impact on decisions. It facilitates rapid sense-making and DM in complex situations where immediate action is required (Epstein, 1994; Stanovich, 1999; Kahneman and Frederick, 2002; Satpute and Lieberman, 2006; Evans and Stanovich, 2013; Gonzalez-Loureiro and Vlačić, 2016; Kahneman, 2017). This capacity for fast, intuitive judgment allows coaches to react swiftly to changes and exploit DM challenges, critical situations, and problems that arise, which can be particularly advantageous in different competitions and training settings. In this respect, System 1 thinking, which is also associated with the use of heuristics in DM processes, can be a source of immediate solutions, insight, and inventiveness, especially if the decision-maker has experience in the specific domain of DM (Klein, 2015, p. 164). On the other hand, System 2 (also System C or analytic system) is characterized by slower, more deliberate, and conscious thinking. It is the system we engage when we need to do complex computations, weigh options judiciously, or when we need to control ourselves (Epstein, 1994; Stanovich, 1999; Kahneman and Frederick, 2002; Satpute and Lieberman, 2006; Evans and Stanovich, 2013; Gonzalez-Loureiro and Vlačić, 2016; Kahneman, 2017). In the context of DM, System 2 is crucial in systematically evaluating the long-term implications of decisions, assessing risks, and ensuring that the choices made align with the overarching strategic goals of the team or individual athlete (Papadakis and Barwise, 1998, p. 127; Elbanna and Child, 2007, pp. 445–446; Bayo and Akintokunbo, 2022, p. 58).

The fundamental cognitive styles (System 1 and System 2) are consistent with the definitions of different authors hierarchically superior to the DM styles (Leonard et al., 1999, pp. 418–419; Spicer and Sadler-Smith, 2005, p. 146; Kozhevnikov, 2007, p. 473; Schoemaker, 2010, str. 22; Dewberry et al., 2013, p. 784), which display themselves at the manifest level as a decision. DM styles, as defined by various authors (Harren, 1979; Rowe and Mason, 1987; Scott and Bruce, 1995; Nygren, 2000), are the result of (1) latent cognitive processes that take place at the level of fundamental cognitive styles and are influenced by the (2) personality traits, (3) biases, (4) amount of stored knowledge, and (5) experience of the decision-maker (coach). Scott and Bruce (1995, p. 820) defined DM styles as a learned response or behavioral pattern of an individual who is faced with a DM situation. They claim that it is not a personality trait but rather a tendency to react in a specific way in a DM situation, whereby the characteristics of the situation itself have a great influence. The authors also state (1995, p. 829) that individual DM styles are not mutually exclusive and that individuals do not rely exclusively on one DM style but use a combination of different DM styles (DM style structure) when making decisions. According to Berisha et al. (2018, p. 3), one of the most frequently used and validated questionnaires for discovering DM styles is the General Decision-Making Style Inventory (hereinafter GDMS) developed by Scott and Bruce (1995). GDSM includes five DM styles: rational, intuitive, dependent, spontaneous, and avoidant. Various studies on different samples (managers, students, the general population, military officers, sports managers, and others) from different countries have numerous authors (Loo, 2000; Thunholm, 2004; Spicer and Sadler-Smith, 2005; Gambetti et al., 2008; Curşeu and Schruijer, 2012; Bavoľár and Orosová, 2015; Alacreu-Crespo et al., 2019; Kolar and Tušak, 2022) confirmed the validity (using factor analysis) and reliability (using Cronbach’s alpha coefficient) of the GDMS inventory as suggested by Scott and Bruce (1995).

Coaches can be understood as highly trained professionals in the field of high-performance sports who possess specific tertiary education and specialist qualifications, making them domain-specific experts with both training and experience in various facets of the sport (Lyle and Muir, 2020, p. 14). Ericsson (2018a, pp. 3-4) wrote that an expert is someone who is highly skilled and knowledgeable in a particular field or someone who is widely recognized as a reliable source of knowledge, techniques, or skills, whose judgments are recognized as having authority and status in public or by their peers. Experts must have long-term and intensive experience with practice and education in a specific field. He goes on to emphasize that expertise refers to the qualities, skills, and knowledge that distinguish experts from novices and less experienced people.

Due to the great diversity in the requirements for achieving excellence and high-level sports results in different sports disciplines, the coaches need to have expertise that properly fits the demands of specific sports disciplines. Coaches must have different domain-specific knowledge related to training in individual sports disciplines if they want to be recognized as experts in a particular sports discipline (Abraham et al., 2006, p. 562). Although it can be argued that coaches have uniform knowledge in some areas related to sports training, regardless of the sports discipline in which they work (e.g., basics of sports training, anatomy, physiology, and so on), differences in knowledge arise mainly from the specific requirements of individual sports disciplines and, in the largest part, from the rules that apply in an individual sport or sports discipline. The competition rules and competition systems of individual sports or sports disciplines determine and define the conditions for achieving success in an individual sports discipline. Namely, the competition rules determine the regular conditions of the competition (time, space, equipment, sports clothes, apparatus, props, and so on) and also what the athlete must do in the sports competition in order to defeat the competitors (run, jump, throw, score a basket, goal, perform a technical element, and so on) and how what it will be shown by the athletes will be evaluated and judged (measured, assessed, scored, ranked, and so on). Competition rules and competition systems, therefore, determine the way sports training is conducted and managed, both in the long-term period (life career, Olympic cycle, annual cycle, and so on) and for each individual training session or competition. Abraham and Collins (2011, p. 378), in their developed nested model, confirmed that there are undoubtedly circumstances in which coaches make (1) deliberative decisions (rational or System 2) based on formal knowledge sources in which the alternative options are relatively clearly identified and also situations where they will use (2) heuristics-based decisions (intuitive or System 1) and use interventional knowledge resources in response to the demands of emerging situations.

From this point of view, it is evident that coaches must possess specialized knowledge and experience in a particular sport or discipline to make informed decisions regarding the career development of athletes. Additionally, coaches exhibit varying decision-making (DM) styles depending on the context: some situations necessitate long-term, deliberate analysis (System 2), while others call for quick decisions based on accumulated expertise and intuitive judgment (System 1).

In the present study, we will deal with the development of the conceptual framework of coaches DM in conventional sports using practical typical cases from gymnastics. According to Matveev’s (1977) classification of sports, which is based on the structural complexity of movements in sports, gymnastics is classified among individual conventional poly-structural sports disciplines, which are characterized by anaerobic energy processes and dominant motor abilities, such as relative strength, coordination, flexibility, and balance. Polystructural sports are characterized by open or semi-open movement structures that are performed in variable external conditions. The conventional character of gymnastics means that all movements (elements) must be performed within a specific movement model (prescribed by experts—convention), which could also be called the ideal movement model (hereafter IMM). Martens (2012, p. 169) defined sport-specific movements as technical skills that are “specific procedures for moving the body to perform the task to be accomplished.” The IMM of the technical skill is defined by a biomechanical model of movement and is predetermined in the evaluation rules (Code of Points: hereinafter: CoP) prescribed by the International Gymnastics Federation. Any deviation from the IMM is considered a rule violation or movement fault, which can be of a technical or esthetic nature. In the CoP, the elements are classified into different difficulty classes depending on the complexity and complicatedness of the movement. The greater the complexity of the movement, the higher the degree of element difficulty and the more points the athlete gains if he successfully performs it in the competition. The evaluation of the performance of athletes in conventional sports disciplines is carried out by judging the implementation of the elements that the athletes present in competitions. They are evaluated by specially trained judges. The evaluation criterion is based on a comparison between the predetermined movement model (IMM) of a technical skill and the actual movement of a technical skill presented by the gymnast. Success in gymnastics is, therefore, defined primarily by the number and difficulty of the elements (technical skills) that the gymnast knows and is able to perform successfully (in accordance with the regulations) in the competition (Kolar et al., 2006, p. 13). The elements learning process consists of didactical methods and techniques of gymnastics elements and is well known as a technical preparation of a gymnast. Technical preparation is a key element of planning, implementation, and control of a gymnast’s overall preparation and also the most important component of a gymnastics coach’s expert knowledge and expertise. In that sense, we can see that the demands of technical preparation represent the basis on which coaches guide the processes of physical, psychological, and all other aspects of a gymnast’s preparation. Based on this, we can conclude that the elements of the learning process are leading a process of the strategic, operational, and tactical DM of gymnastics coaches.

All the above-mentioned specifics of sports training in general and particularly in conventional sports have a significant impact on the management and DM processes of coaches and thereby significantly co-shape their DM style’s structure and DM behavior. This study aims to (1) review relevant DM theories and coaching models and (2) develop a conceptual framework for coaching DM in conventional sports, focusing on gymnastics.

## 2 Literature review

Despite the growing recognition of the importance of coaches’ DM in the training process, characterized by the need to adapt or align DM behavior with the specific or unique requirements of the athlete and the chosen sport (Harvey et al., 2015, p. 152), research in the field of sports coaching theory continues to reveal a lack of empirical insights into coaches’ DM styles. Many variables affect the implementation of the coaching process: (1) team or individual sport, (2) age of athletes, (3) ability of athletes, (4) coaching philosophy, (5) understanding of the coaching process, (6) coaching environment, and (7) level of effectiveness (Nash and Collins, 2006, p. 467). These factors suggest that an expert coach is someone capable of making appropriate decisions within the constraints of their coaching practice, reinforcing the belief that coaching is fundamentally a cognitive activity (Lyle, 1999).

### 2.1 Sports coach’s leadership styles in contexts of DM behavior

There are quite a few studies in which the authors dealt mainly with the leadership styles and behaviors of sports coaches (Jin et al., 2022, p. 1; Jawoosh et al., 2022, p. 1115) and applied the findings to their DM behavior in various situations and contexts. In the field of studying the behavior of coaches in gymnastics, a study conducted by Côté et al. (1995) often cited that the behavior of coaches is characterized by the integration of performer, performance, and contextual factors. In the study, the authors present the concept of mental models through which coaches organize their knowledge, which is a fundamental prerequisite for an expert DM (Côté et al., p. 10). In addition, Chelladurai and Arnott (1985) describe three different leadership styles of the coach, including the autocratic, participative, and delegative styles, and suggest that the best decision style in any circumstances relies on the configuration of the attributes of the problem. They argue that situational elements were the major factors attributed to coaching style rather than the traits or personality of the coach. Kaya (2014, p. 335) emphasized that the situational leadership style is the most common style for coaches and outlined four subdomains of this style: telling, selling, participating, and delegating. He argues that before a leadership style is implemented, the level of an athlete’s acceptance and readiness should be assessed to determine the coach’s best-fit leadership style. In these studies, authors mainly determine coaching leadership style, which can be categorized as task factor, decision factor, and motivational factor (Jawoosh et al., 2022, p. 1115).

The decision factor can be understood as the manner in which the DM process is implemented, primarily observed through the interaction between the coach and the athlete. This interaction typically falls into one of three styles: autocratic, participative/democratic, or delegative/laissez-faire (Feu et al., 2010). It does not focus on the cognitive processes coaches use to make decisions but rather on how they involve athletes in the process. Coaches with a dominant autocratic style make decisions entirely on their own. Those with a prevalent democratic leadership style engage in the entire DM process collaboratively with the athlete. In contrast, coaches with a laissez-faire leadership style make judgments independently but seek the athlete’s consent and agreement before finalizing decisions.

Elderon (2020) emphasized that a good coach adapts their style to the situation, often favoring the participative approach in contexts that require learning, DM, and problem-solving. Similarly, Sherman et al. (2002, p. 390) recommend flexible use of DM and coaching styles and adapting them to match those to which the athlete is receptive. Marshall (2006, p. 160) supports the argument that successful coaching of high-level athletes involves a much more consensual process than the do-as-I-say approach, while Kolar et al. (2006, p. 18) wrote that the relationship between (1) the athlete’s biological and sports development phase and (2) the coach’s leadership style changes from more autocratic in the period of youth to more participative in the period of growing up and maturing. The authors of these studies mostly focus on determining the coach’s leadership style, which is mainly reflected in the level of the athlete’s involvement in DM processes, and try to find correlations between the leadership style and the athlete’s motivation (Charbonneau et al., 2001; Andrew and Kent, 2007; Wu et al., 2014; Jin et al., 2022), satisfaction (Andrew and Kent, 2007; Andrew, 2004; Kim and Cruz, 2016; Jin et al., 2022; Jawoosh et al., 2022), team cohesion (Jowet and Chaundy, 2004; Kim and Cruz, 2016; Nascimento-Júnior et al., 2018), performance (Charbonneau et al., 2001; Moen et al., 2014; Jawoosh et al., 2022), or burnout (Harris, 2005).

Studies of the leadership styles of sports coaches also have another important message. They show that a more participative leadership style allows athletes greater autonomy in expressing their opinions and concerns, thereby ensuring coaches obtain more relevant feedback about the impact of training on the development of athletes’ careers (Moen et al., 2014; Elderon, 2020) and is associated with positive outcomes (Lyle and Muir, 2020, p. 8). Voight (2002, p. 44) argues that based on the feedback the coaches receive, coaches can effectively implement the strategies or personal skill development of the athletes. For example, Dunn (2006, p. 3) states that feedback is a critical part of the learning process in the DM for coaches and, according to Hodges and Franks (2002, p. 793), a critical factor in coaching success and decisions.

### 2.2 Natural decision-making paradigm in the DM processes of sports coaches

Abraham and Collins (2015, p. 1) report that there has been recently growing interest in using the naturalistic decision-making (hereinafter: NDM) paradigm and recognition decision-making (hereinafter: RPD) model to examine and understand DM of sports coaches in time-limited situations. The NDM approach (Klein, 2008, 2015) is an alternative to the normative rationalistic DM process approach, whose main orientation is that decision-makers in natural settings rely heavily on expert intuition. Mosier et al. (2018, p. 453) claim that NDM researchers promoted models more suitable to explain the rezoning of experts in domains characterized by dynamic conditions, time pressure, uncertainty, high stakes, multiple players, and organizational constraints. Their studies suggest that DM in these domains could not be reduced to a single moment of choice after all the facts had been analyzed but rather as immediate actions with imperfect knowledge. NDM theory assumes that specific-domain experts rarely consider more than one option at a time but instead expend most of their effort on situation assessment. Authors wrote that with experts, decisions come from recognizing and/or making sense of the situation, which is consistent with Simon’s (1992, p. 155) claim that the (expert) intuition is nothing more and nothing less than recognition. Lyle and Muir (2020, p. 16) summarized coaches’ DM in NDM settings and elaborated that coaches will scan and attend to key attractors (domain-specific stimuli that carry particular ‘weight’ as catalysts for action) in an ever-evolving environment (athletes, results, opposition, crises). This can lead to a problem-framing response if the target challenge threshold is exceeded. Otherwise, the routine activity (or inactivity) will continue. A quick situational analysis links the problem to a potential course of action. Experts use their experiences to focus their perceptions on salient features, to recognize situations as typical, and to choose the most appropriate option for action. The RPD model (Klein et al., 2010, pp. 193–194) postulates that experts’ DM is a recognition process and suggests that the vast majority of expert decisions in naturalistic settings are so-called (1) prototype decisions when experts encounter typical situations, recognize a match to a prototype, and the prototypical scenario guided by experience tells them how to proceed and implement a course of action, without ever considering any of the other options at the decision point. Furthermore, they reported that there is a small share of decisions in naturalistic settings when experts identify two or more ways of accomplishing a goal and then make the selection based on a single dimension or only a few dimensions, and that way, consciously compare options to arrive at a decision. This decision type they named as (2) deliberated. Even if this category describes a standard way that DM is studied in laboratories (normative theory), as the authors wrote, they did not see any evidence that this decision type used the classical normative DM approach. The mentioned two decision types are related to situations known to experts, while the third decision type is related to unfamiliar situations. They argue that when experts faced an unfamiliar situation, they had to creatively generate or construct the possible options. This decision type is called (3) constructed and is mainly connected with constructing a new, unique solution with the use of existing experience in an innovative way (Klein et al., 2010, p. 203). The authors emphasize that the advantage of the RPD model is that it provides the decision-maker with a course of action at every point. The decision-maker begins with an initial option, and if a response is called for, this will be executed. If there is time for some evaluation, it will be examined, accepted, improved, or rejected for a second option, which then becomes primed for implementation. Instead of comparing and evaluating several options, which is a time-consuming process, decision-makers must rely on their experience and ability to quickly recognize the causal dynamics of situations as a way of generating effective options and evaluating them (Klein et al., 2010, p. 205). They also explained that the RPD model is not simply about intuition but is a blend of intuition (the prototype matches, which today would be described as pattern-matching) and analysis (the mental stimulation) (Klein et al., 2010, p. 207).

Bossard et al. (2022, p. 1) in their study perceive that there is an extended number of studies where different authors use the RPD model to find out the DM behavior of athletes (Macquet and Fleurance, 2007; Macquet, 2009; Kermarrec and Bossard, 2014; Macquet and Kragba, 2015; Milazzo and Fournier, 2015; Le Menn et al., 2019; Fortin-Guichard et al., 2021) and expert coaches (Abraham and Collins, 2015; Harvey et al., 2015; Collins et al., 2016; Collins and Collins, 2016; Ashford et al., 2020) from different sports in natural settings. Findings suggest that coaches have an initial wish to engage in RPD-type behavior and have the capacity to be ‘expert’ but may not use this capacity unless forced to do so (Abraham and Collins, 2015, p. 1). Also, Harvey et al. (2015, p. 152) stated that NDM can offer a suitable framework to apply to coaches’ DM behavior. Collins et al. (2016, pp. 5–6) add that there are considerable variations, both between coaches and between sports, in the perceived frequency of intuitive DM use (RPD model). They found out that in all cases, coaches acknowledged the need for careful planning across all elements of their work, where the intuitive aspects of the coaches’ DM emerged differentially across the macro (planning stage) and micro (implementation stage) processes of the training session. Moreover, Richards et al. (2017, p. 73) argue that the DM process is complex and multifactorial, where the crucial underpinning for the efficient application of the coach’s tactical knowledge is the use of a slow, deliberate, and reflective examination of the process. In the field of the sports training process, there are certainly many situations in which coaches make decisions consistent with the NDM paradigm and the use of the RPD model, but as Kahneman and Klein (2009, pp. 524–525) point out, there are three fundamental conditions for valid intuitive reasoning. The environment within which the reasoning takes place (1) must be orderly, and there (2) must be the possibility for the decision-maker to learn the rules of its orderliness and (3) have adequate feedback about his thoughts and actions. Only if all conditions are met at the same time will the associative memory (stored tacit knowledge or experience) be able to recognize the circumstances and produce quick and accurate decisions.

### 2.3 Professional judgment and decision-making concept of sports coaches DM

The introduction of the NDM paradigm and the PRD model into the field of the sports coaching process has enabled a better understanding of how coaches deal with the complexity, immediate crises, and uncertainty of the training process, and it should be understood as a mechanism through which it is possible to explain and understand how coaches operationalize DM in dynamic micro-moments of coaching intervention. However, as Lyle and Muir (2020, p. 21) noted, there is relatively little recognition-based non-deliberative DM, and in this context of semi-deliberative DM, extreme models of NDM are useful but not a perfect solution. It is argued that most DMs have an element of deliberation apart from immediate crises. Similarly, Wilson and Kiely (2023, p. 4) contend that, within sporting contexts, some DM tasks are mundane and routine and hold very high validity. In such contexts, effective heuristics, or simple rules of thumb, provide fast, effective, and accurate decision-making guides if the aforementioned conditions are fulfilled (Kahneman and Klein, 2009, p. 524). However, the sporting environment can sometimes not give the coach the opportunity to learn from it and have solid feedback on his actions and can be messy, which can be described as volatile, uncertain, complex, and ambiguous (hereinafter VUCA) (Codreanu, 2016, p. 31). In these environments, outcomes are highly unpredictable with no coherent, identifiable, learnable patterns and predictions and, accordingly, have low to zero validity (Wilson and Kiely, 2023, p. 4). Coaches tasked with making decisions in VUCA-like environments cannot rely on intuition-informed guidance and the NDM model because they can be misled into wrong conclusions, judgments, and decisions (Kahneman and Klein, 2009, pp. 524–525).

In addition, Abraham and Collins (2011), along with Martindale and Collins (2007, 2013), argue that the classic decision-making model (normative model, hereafter CDM), like the naturalistic decision-making (NDM) paradigm, has limitations. They introduced the concept of professional judgment and decision-making (PJDM) as a comprehensive framework for understanding and facilitating the complex decision-making behaviors of sports coaches. By integrating the principles of CDM and NDM into PJDM, the authors propose that coaches make decisions along a continuum, ranging from logical and rational choices to intuitive, experience-based decision-making. This aligns with the dual-process model of decision-making, as conceptualized by various scholars (Taggart and Valenzi, 1990; Langley et al., 1995; Miller, 2008; Kruglanski and Gigerenzer, 2011; Dhami and Thomson, 2012).

Collins et al. (2016, p. 2) further describe the concept of nested decision-making, an application of PJDM to coaching. They suggest that higher-order, longer-term (strategic) decisions should be made in a more deliberative manner (reflective of CDM), while immediate, in-session (operative) decisions are often short-term and almost intuitive (reflective of NDM). This “nesting” of intuitive/operative decisions within deliberate/strategic decisions ensures that short-term choices align with and contribute to achieving long-term goals. Such an approach, exemplified in sports coaching, can enhance the implementation of planned long-term strategies while addressing immediate situational demands.

One of the first authors to draw attention to the complementary use of both DM concepts was Simon (1987, p. 63), who claims that an effective decision-maker cannot exclusively use an intuitive or rational DM style when making decisions but that they must be able to use both concepts of DM, depending on the problem situation. This means that they must be flexible in the use of concepts, and he states that intuition (NDM) is also an analytical cognitive process (CDM), which is otherwise unconscious and in which, at this level, decision-makers synthesize experience and acquired knowledge in future decisions. The PJDM process empowers coaches to effectively utilize their skills by designing tailored teaching strategies, planning programs, linking sessions, and adapting to individual athlete needs. Through this adaptive approach, PJDM not only fosters creativity and adaptability in coaches but also enhances the coaching process. The model emphasizes the importance of reflection, storytelling, simulation, and the coach’s beliefs and knowledge in making optimal decisions across different levels of coaching: micro, meso, and macro. Ultimately, the PJDM framework is supposed to serve as a holistic tool for enhancing coaching performance and facilitating a dynamic and effective coaching environment (Collins et al., 2016, p. 2).

### 2.4 DM styles as a concept of DM behavior of sports coaches

The PJDM model, therefore, assumes that the DM process of coaches, depending on the (1) problem situation and the (2) goal of the decision, takes place both within System 1 (specific domain expert intuition) and within System 2 (bounded rational analytical process). The latent use of different cognitive styles (System 1 and 2) used by coaches in their DM behavior can be detected through manifested DM styles that shape the coach’s DM behavior, which means that cognitive styles can be understood as hierarchically superior cognitive structures to the DM styles (Leonard et al., 1999, pp. 418–419; Spicer and Sadler-Smith, 2005, p. 146; Kozhevnikov, 2007, p. 473; Dewberry et al., 2013, p. 784). Manifest DM behavior and the structure of coaches’ DM styles can be observed and measured with questionnaires that achieve adequate reliability and validity. A review of the existing literature shows two relevant articles where the DM behavior of coaches was determined by using the GDMS questionnaire. Giske et al. (2013) investigate soccer coaches’ DM styles in relation to elite and non-elite coaching experience and level of playing history. The results of their study show that soccer coaches mostly use rational or intuitive DM style and almost no avoidant DM style and that coaches with more expertise in specific domains of coaching statistically significantly use more rational and intuitive DM styles than non-experts. Additionally, coaches with elite-level player experience also show statistically significantly greater use of intuitive and rational DM styles than coaches without that experience (Giske et al., 2013, p. 695). The second study, which was conducted by Noh et al. (2018), explores the relationship between soccer club coaches’ DM style, basic psychological needs, and intention to continue to exercise. This study’s results showed that coaches’ rational and intuitive DM styles have a positive effect on the participants’ basic psychological needs, while coaches’ dependent and avoidant styles have a negative effect on their basic psychological needs. Furthermore, this study also revealed that coaches’ rational and intuitive DM styles have a positive effect on sports participants’ intention to continue to exercise, while coaches’ avoidant style has a negative effect on their intention to continue to exercise (Noh et al., 2018, p. 10). In both studies, the structure of DM styles within all formed samples (entire, experts, non-experts…) was the same. Coaches demonstrate the highest proportion of use of both functional DM styles (1) rational and (2) intuitive, followed in order by the so-called non-functional DM styles, (3) dependent, (4) spontaneous, and (5) avoidant DM style. The use of functional DM styles in DM processes generally leads to correct and effective decisions, while the increased presence of non-functional styles in the overall DM style structure of coaches could indicate the risk that their DM behavior often leads to negative results and erratic decisions (Mitchell et al., 2011, pp. 693–694; Faletič and Avsec, 2013, p. 133).

### 2.5 Other concepts of sports coaches’ DM behavior

The literature review also provides other aspects and classifications of coaches’ DM. A recent study by Post and van Gelder (2023, p. 25) provides a typology of seven main types of decisions made by coaches based on the types of cognitive processes involved in making those decisions. The authors also create a simple framework that breaks decision-making down into four phases: (1) sense-making, (2) option generation, (3) option evaluation, and (4) selection (Post and van Gelder, 2023, p. 34). Except for the “snap” type decision (1st type), which can be fully understood as a System 1 decision, i.e., intuitive (RPD model), and the “pros and cons” type (7th type), which is defined as a classic analytical-rational type of DM using System 2 (CDM model), for other types, the definition of the used cognitive style when making a decision is unclear. For example, in the “simulation” (second type), “rule” (third type), and “analogy” (fifth type) types of decisions, coaches may arrive at decisions based on the System 1 process since the “simulations,” “rules,” and “analogies,” as long as they are previously known and/or experienced, are part of the expert’s tacit knowledge stored in memory. In these cases, the DM process or judgment takes place in an unconscious analytical process (Simon, 1987, p. 63) (generating and evaluating of options phase), where the coach, based on the recognized situation (sense-making phase), comes to an appropriate solution or decision (selection phase) effortlessly since unconsciousness and effortlessness are the fundamental determinants of the operation of System 1. The described process is fully consistent with the RPD model and the understanding of the operation of expert intuition. However, coaches can perform all three types of DM also in the analytical-rational process of System 2, as long as in the phases of generating and evaluating options, they generate and evaluate them in a conscious regime by investing effort, and it is possible that they do this exclusively by using active thinking or searching for information in the external environment. The “metaphor” (4th type) and “story” (6th type) types of decisions can be understood in the same way from the perspective of the cognitive style used, depending on whether the “metaphors” and “stories” are recognized unconsciously (System 1) or through conscious (System 2) search for an appropriate one. However, there is one distinction between these two types of coaching decisions. As the authors wrote (Post and van Gelder, 2023, p. 25), “metaphors” and “stories” are more useful for describing and understanding the situation (sense-making phase) than for generating and evaluating options or making a decision. Moreover, it is also important for coaches to understand that “metaphors” and “stories” can also produce a strong framing or representative bias that can mislead coaches in judgment and creating decisions (Tversky and Kahneman, 1974, 1981; Hammond et al., 2013).

## 3 Research design and method

To design the research, we used the methodology supported by Gilson and Goldberg (2015, p. 128) when they explained the writing of a conceptual paper that provides a bridge or connection between different concepts and scientific disciplines. The method approach was the “model paper,” which seeks to build a theoretical framework that predicts relationships between concepts. A model paper identifies previously unexplored connections between constructs, introduces new constructs, or explains why elements of a process lead to a particular outcome (Jaakkola, 2020, p. 24).

In that manner, based on a review of representative sources, (1) a general understanding of sports coaching and the central role of the coach in that process is conceptualized, (2) the importance of coaches DM in the training process is defined, (3) the DM process in general and attributes of the dual model of latent cognitive styles and manifest level DM styles are described, (4) specific-domain characteristics of conventional poly-structural sport disciplines are explained, and (5) the relevant DM theories and previous development models and concepts of coaching DM are reviewed and discussed. Based on the steps described in research (6), a conceptual framework will be built that establishes appropriate relationships between various decisions that coaches make in a training process and different theoretical concepts related to DM in general and coaches DM in particular. The developed conceptual framework is presented from substantive and process points of view. A conceptual framework will be built for coaching DM in conventional poly-structural sports; however, the practical examples and applications will be taken from gymnastics as one of the most typical sports with that kind of structural characteristics.

## 4 Development of a conceptual framework

**Figure 1:**
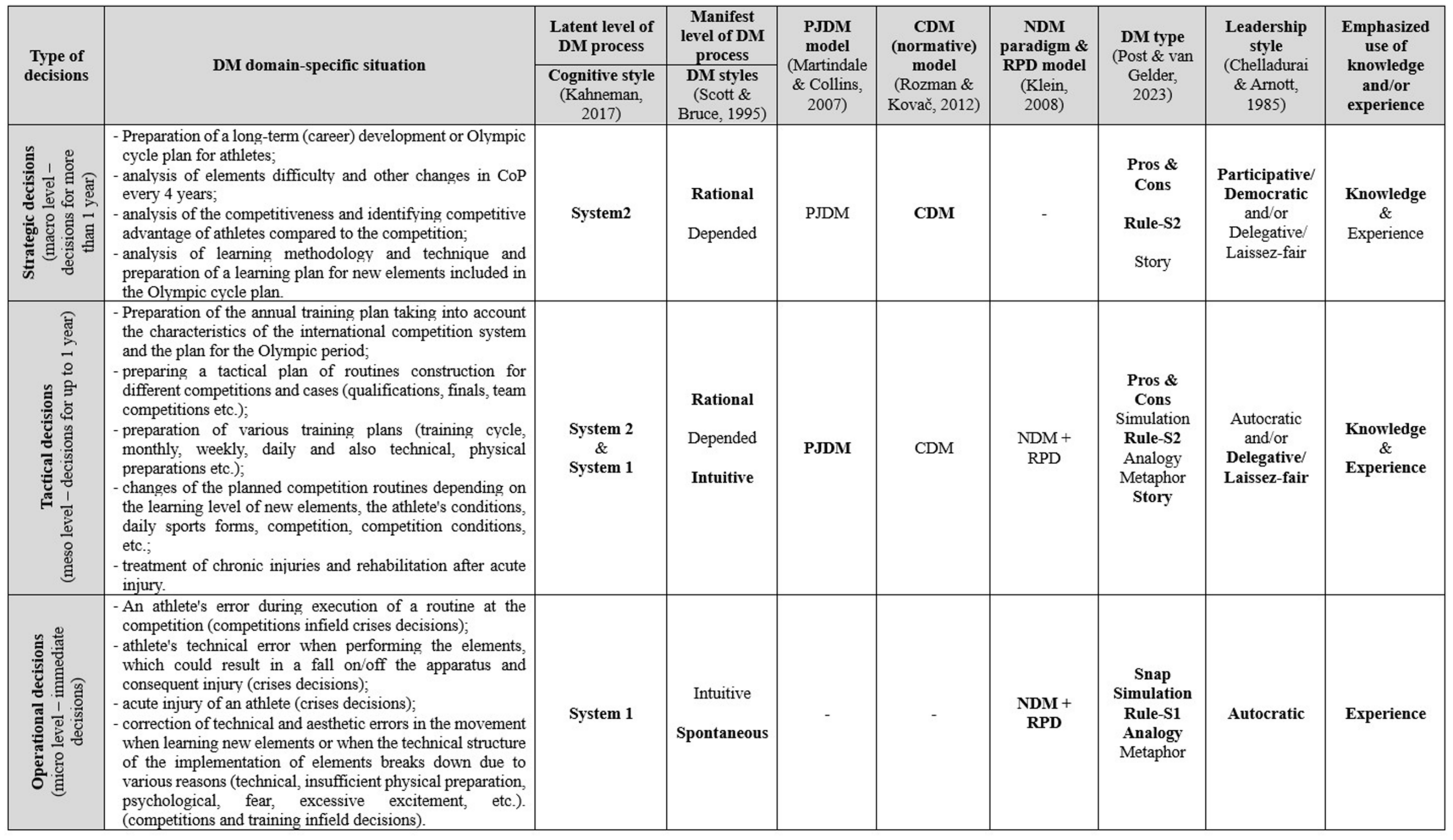
Conceptual framework of gymnastics coaches DM, depending on the type of decisions. Bolded words indicate the predominant use of that style/model/type/concept in a particular type of decision.

Figure 1 shows the developed conceptual framework of coaches’ DM in conventional sports, where we integrate different concepts of coaches’ DM behavior (Chelladurai and Arnott, 1985; Martindale and Collins, 2007; Post and van Gelder, 2023) with general constructs of DM theories (Scott and Bruce, 1995; Klein, 2008; Rozman and Kovač, 2012; Kahneman, 2017).

The conceptual framework is broadly divided into distinct types of decisions that coaches make, categorized based on three primary criteria: (1) the expected time frame of validity, (2) the time impact, and (3) the level of urgency of the decisions. Accordingly, coaches’ decisions are classified into three levels: strategic (macro), tactical (meso), and operational (micro). Strategic decisions have a duration and validity of more than 1 year; tactical decisions are valid for up to 1 year, and operational decisions require immediate enforcement.

This division based on time frames, as depicted in Figure 1, aligns with the framework proposed by Richards et al. (2012, p. 3). In their cyclical model of decision-making (DM) in sports, the first phase primarily employs the CDM (normative) model to develop a general strategy for team DM behavior (long-term DM). The second and third phases focus on creating and implementing a tactical preparation plan (middle-term DM), while the fourth phase predominantly utilizes the NDM (intuitive) model to implement the plan during sports competitions (immediate DM).

A notable feature of Richards et al.’s model is its emphasis on feedback loops. The on-field implementation of the tactical plan (third phase) provides critical feedback to refine the off-field theoretical tactical preparations (second phase), ensuring alignment with the overall strategy developed in the first phase. This cyclical interaction reinforces the dynamic and iterative nature of effective decision-making in sports coaching.

For each proposed type of decision, some typical specific-domain situations are also added to the conceptual framework (column 2), which represent problem situations that coaches judge in DM processes and make appropriate decisions for. The concept of leadership styles (column 9) and the competency model (column 10) are also added. The concept of leadership styles of coaches shows the most desirable or suggested leadership styles for each type of decision, i.e., those styles, the use of which for a particular type of decision would have the greatest potential for success and effectiveness in the formulation and implementation of decisions. The competency model reveals two crucial fundamental DM competencies, where knowledge is understood as explicit knowledge or formal theoretical knowledge acquired by coaches in the process of formal or/and informal education or individual theoretical learning, while experience is understood as domain-specific implicit or tacit knowledge that is intuitive and acquired through practical experience (Nash and Collins, 2006).

While explicit knowledge primarily refers to theoretical understanding, implicit knowledge (experience) encompasses both theoretical and practical elements. Theoretical implicit knowledge can manifest as the application of rule-based decisions (“rule-S1”), such as identifying and addressing an athlete’s error during the execution of an exercise in competition. On the other hand, practical implicit knowledge can take the form of physical or motor intervention by the coach, such as stepping in to protect the athlete and prevent an acute injury caused by a movement error, such as a fall on or from the apparatus.

In the developed conceptual model, various forms of DM behavior of coaches are foreseen for different types of decisions, with these behaviors intertwined at the manifest level depending on the specific situation that requires a coach’s decision. One notable DM style not explicitly addressed in the conceptual framework is the avoidant DM style. This style manifests as a tendency to avoid making decisions or displaying indecisiveness, which ultimately does not lead to resolving the perceived decision problem. While avoidant behavior can be observed at the manifest level, its inclusion in the overall structure of coaches’ DM styles highlights a potential limitation or challenge in effective decision-making within the coaching process (Scott and Bruce, 1995, p. 820). Moreover, the avoidant DM style is characteristically associated with negative or pathological personality traits. These include aggressiveness (Faletič and Avsec, 2013, p. 139), neuroticism (Riaz et al., 2012, p. 100; Faletič and Avsec, 2013, p. 139; Narooi and Karazee, 2015, p. 313), emotional instability (Alacreu-Crespo et al., 2019, p. 746), unsociability (Avsec, 2012, p. 215), unconscionability (Narooi and Karazee, 2015, p. 313; Bayram and Aydemir, 2017, p. 911; Alacreu-Crespo et al., 2019, p. 746; El Othman et al., 2020, p. 7), anxiety (Avsec, 2012, p. 215), poor self-esteem (Thunholm, 2004, p. 940), and higher stress levels, poorer sleep, and depression (Leykin and DeRubeis, 2010, p. 510; Schoemaker, 2010, pp. 57–58; Allwood and Salo, 2012, p. 34; Bavoľár and Orosová, 2015, p. 119). Therefore, avoidant DM style is defined as completely dysfunctional or “pathological,” the use of which does not lead to a solution to the DM problem. Regardless of the level of knowledge and/or experience a coach has, a coach with a high value of the presence of an avoidant DM style in his individual DM style’s structure will not be able to make any decision, or the made decisions will be completely dysfunctional. Such a coach must not be assigned any DM role at any point in the overall training process. In addition, if a coach with a high presence of avoidant DM style had DM authority or power and also scored high in a (1) rational DM style scale, he is likely to require never-ending analyses with no conclusion. If he had a high value of (2) dependent DM style, he would probably use his colleagues to transfer responsibility for decisions to them, and if he had a high level of (3) intuitive or (4) spontaneous DM style, he would make any decision regardless of its consequences because he is unable to think about them.

### 4.1 Strategic-type decisions

Strategic-type decisions involve committing substantial resources, setting precedents, and initiating a cascade of lesser (tactical-type) decisions that significantly influence the success or failure of long-term intentions and goals (Dean and Sharfman, 1996, pp. 379–380). According to the conceptual framework, strategic DM should primarily align with the (normative) CDM model and “pros and cons” DM types (Post and van Gelder, 2023, p. 25). At the latent level, this process is driven by a rational-analytical cognitive style (System 2), mainly characterized by the extensive use of decision-makers’ knowledge. Coaches who exhibit a dominant rational DM style and are willing and able to seek confirmation and consensus from a broader circle of domain-specific and interdisciplinary experts (dependent DM style) are more likely to succeed in making effective strategic decisions. This is consistent with findings that rationality in strategic DM is positively associated with decision effectiveness, quality, and implementation success (Papadakis and Barwise, 1998, p. 127; Elbanna and Child, 2007, pp. 445–446; Al-Hashimi et al., 2021, p. 913; Bayo and Akintokunbo, 2022, p. 58). Within the conceptual framework, rational-analytic reasoning (System 2) aligns with Simon’s theory of bounded rationality, which recognizes internal and external limitations on decision-makers (Simon, 1979, p. 502). This reasoning is both procedural (logical and sequential DM processes) (Simon, 1976, p. 131; Alkaraan and Northcott, 2013, p. 126) and extended (incorporating external expertise) (Secchi, 2010, pp. 142–143). Musso and Francioni (2012, p. 281) emphasize that strategic rationality—the capacity to follow a systematic (procedural rationality) process to reach well-considered objectives—is a critical competency, though often constrained by the limited cognitive capabilities of decision-makers.

Within the conceptual framework, rational-analytic reasoning (use of System 2) aligns with Simon’s theory of bounded rationality, which recognizes internal and external limitations on decision-makers (Simon, 1979, str. 502). This reasoning is both procedural (logical and sequential DM processes) (Simon, 1976, p. 131; Alkaraan and Northcott, 2013, p. 126) and extended (incorporating external expertise) (Secchi, 2010, pp. 142–143). Musso and Francioni (2012, p. 281) emphasize that strategic rationality—the capacity to follow a systematic (procedural rationality) process to reach well-considered objectives—is a critical competency, though often constrained by the limited cognitive capabilities of decision-makers to make rational decisions (bounded rationality). The importance of using an extended rational-analytical approach to the formulation of strategic-type decisions is that these decisions tend to involve the distillation of complexity into a single path forward (Kahneman et al., 2019, p. 67). From the point of view of DM types proposed by Post and van Gelder (2023, p. 25), the strategic types of decisions in conventional sports (gymnastics) are characterized by the extended use of rule-based decisions, where, in this framework, it is not a case of simple heuristics of the “if-then” type decisions (“rule-S1”) but for broad theoretical knowledge of the international competition system and CoP (“rule-S2”). This knowledge forms the basic substantive background of all strategic decisions in gymnastics, so it is important that expert judges are part of the teams that create this type of decision because they have proven and verifiable knowledge of the provisions of the CoP. As we have already emphasized, it is important that coaches at this level of DM also demonstrate a significant share of the dependent DM style, as this demonstrates the intention of a broader and open dialog about strategic directions in the process of development of athletes’ results and sports discipline in general. For this reason, the use of a participative/democratic leadership style is the most suitable for creating and making strategic decisions, as it enables the extension of a rational approach to DM. However, caution should be exercised when interpreting the increased presence of a dependent DM style since it indicates a positive contribution to DM processes only if the dominant style in the coach’s individual DM style structure is a rational DM style. If an individual coach’s DM style structure strongly expresses the avoidant DM style in connection with the dependent DM style, then this indicates that the coach does not seek additional information to expand the rationality of the decision but tries to transfer the responsibility for making the decision to others (Harren, 1979, p. 121; Faletič and Avsec, 2013, p. 133; Giske et al., 2013, p. 696; Fischer et al., 2015, p. 525).

### 4.2 Tactical-type decisions

**Figure 2:**
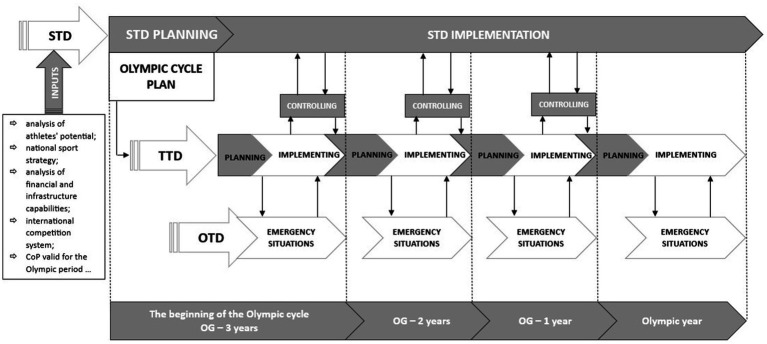
Process perspective of the conceptual framework of gymnastics coaches DM (application for the planning and implementation of the Olympic cycle period). STD, strategic-type decisions; TTD, tactical-type decisions; OTD, operational-type decisions; OG – x year, x years to the year of the Olympic games.

The development of tactical-type decisions is already part of the strategic-type decisions implementation process (Figure 2), and as such, they are no longer part of the strategic DM process in a narrow sense but of the newly established tactical DM process. The implementation of selected strategic-type decisions is the phase that comes when a strategic-type decision is already adopted, and if they are not implemented, they will not have any impact on the athlete’s result development process. Formulating a tactical-type decision is a process where coaches and other experts make action plans to implement strategic-type decisions annually and for different training cycles (preparation cycle, pre-competition cycle, and competition cycle) and time-specific periods (monthly, weekly, and daily). These types of decisions must fully support the realization of strategic-type decisions, which means that all forms of athlete preparation (technical, physical, psychological, and so on) must be directed and consistent with the strategic goals that arose based on strategic-type decisions. In a practical sense, this means that all training and competition activities must be aimed at establishing all the necessary conditions so that at the end of a strategic period (e.g., the Olympic cycle), the athletes will be able to perform routines (technical preparation) with a planned degree of difficulty and technically and esthetically flawless execution. This DM process can be best described with the use of.

Martindale and Collins’ (2007, 2013) PJDM holistic model integrates decisions informed by both the (normative) CDM model and the (intuitive) NDM model. Within this framework, decision-making types such as “pros and cons,” “rule-S2,” and “story” (Post and van Gelder, 2023, p. 25) are identified as having the best fit. The model highlights the dynamic interplay between System 1 (intuitive) and System 2 (analytical) cognitive styles, as described by Kahneman (2017) and Calabretta et al. (2017), underscoring how these dual processes interact to shape decision-making in complex and context-dependent scenarios. Tactical-type decisions arise with the use of decision-makers’ knowledge in combination with the support of their practical experience. Coaches, during this process, manifest both the rational (mostly when planning) and intuitive (mostly when implementing plans) DM style, where the presence of a (rationally) dependent DM style is desirable, as it enables coaches to participate in wider teams of experts, expand their rationality, and control cognitive biases, which may appear mainly in the initial unconscious solutions of the intuitive-experience System 1 (Norman et al., 2018, p. 334). From this point of view, in the conceptual framework (Figure 1), the delegative/laissez-faire type of leadership is proposed as the most suitable type of leadership when dealing with this type of decision, where coaches make decisions independently, based on knowledge and experience, but the validity of the decision is also checked with others involved in the process (also athletes). This process is also characterized by the fact that coaches create plans for various “unpredictable” situations and thus “if-then” (“rule-S1”) responses in the event that these situations occur during the implementation of training and/or competitions (an error in the routine at the competition, injury or illness of an athlete, change of competition, and so on). By pre-preparing this type of response, trainers enrich the specific-domain knowledge base, which will easily be found in the associative memory by System 1, and unconsciously and quickly formulate a solution and appropriate action (RPD model) for an urgent “unplanned” event (operational-type decision). Tactical-type decisions are related both to the theoretical planning and practical implementation of the sports training process and are the most important decisions for effectively implementing strategic-type decisions. This decision needs to be controlled on a level of training (level of athlete’s preparation) and on a level of competition’s success (level of competitional realization). Hickson et al. (2003, p. 1803) concluded that the way decision implementation is managed appears to be vital for decision success.

### 4.3 Operational-type decisions

Operational-type decisions take place and are enforced within the framework of the implementation of the tactical-type decision process (Figure 2). They most often act as a corrector of unforeseen events or events that differ from the planned ones. These events often create intervention situations within which DM is urgent and time-constrained. This type of decision mostly arises suddenly in accordance with Klein’s (2008) NDM paradigm and RPD model and a “snap” DM type, which may be supported by the unconscious use of “simulations,” “rule-S1,” and “analogy” DM types (Post and van Gelder, 2023, p. 25). For the operational type, decisions are at the latent level responsible for the intuitive-experiential cognitive style (System 1), where the DM process is mainly characterized by the use of decision-maker experiences and the amount of accumulated domain-specific tacit knowledge. When an emergency arises, the coaches must demonstrate an autocratic leadership style; because of time constraints and the urgency of response, they do not have time to explain the decision to others and negotiate it. These decisions made in a so-called naturalistic environment can only be valid if they are made by an experienced expert, i.e., a coach with a sufficient amount of accumulated tacit knowledge that was acquired in a specific domain environment, whose rules of order he had the opportunity to learn on the basis of frequent and correct feedback information (Kahneman and Klein, 2009, pp. 524–525). Novices or coaches with insufficient experience cannot make these decisions, as they would represent mere guesses subject to various cognitive biases, and their validity would depend solely on luck. The autocratic approach in enforcing such decisions is based on the athlete’s acceptance, trust, and faith in the coach’s knowledge and experience (Kaya, 2014, p. 335). Therefore, the authority of the coach must be built on the basis of professional knowledge (expert-based authority) and not only on the basis of the superior position and the organizational role that the coach has in relation to the athlete. The operational-type decisions are shown on a manifest level in the form of an intuitive and mostly spontaneous DM style. Spontaneous DM style has been repeatedly found to have a statistically significant positive association with intuitive DM style (Scott and Bruce, 1995, pp. 827–828; Thunholm, 2004, p. 938; Spicer and Sadler-Smith, 2005, p. 141; Geisler and Allwood, 2018, p. 420, p. 65) and avoidant DM style (Scott and Bruce, 1995, p. 829; Spicer and Sadler-Smith, 2005, p. 141; Hariri et al., 2014, p. 293; Reyna et al., 2014, p. 36; Bayram and Aydemir, 2017, p. 5). In the case when a good feeling (gut feeling or feeling of rightness) about the initial solution (System 1) is connected with a sense of urgency to make a decision, the decision will manifest itself in the form of a spontaneous DM style, which in this case would still work as a functional style, and its use would lead to a positive outcome (Faletič and Avsec, 2013, p. 133). In this case, it can also be understood in accordance with Thunholm’s (2004, p. 941) definition of the spontaneous DM style as a high-speed, intuitive DM style used in DM situations that are under time pressure. However, when the sense of urgency inhibits the negative feeling (gut feeling) about the initial solution and the coach makes a quick decision anyway, such use of a spontaneous DM style can be classified in the category of dysfunctional DM styles, which often lead to negative outcomes and erratic decisions (Mitchell et al., 2011, pp. 693–694; Faletič and Avsec, 2013, p. 133). Coaches who adopt the spontaneous DM style essentially aim to avoid a non-decision situation, which characterizes the avoidant DM style, by accepting any kind of decision rather than thoroughly evaluating their options.

## 5 Discussion

Sports coaching in conventional sports is a complex process in which the main role is played by coaches, who have to make different decisions throughout the process and enforce them with different approaches and leadership styles. Some decisions are the result of deep thinking based on the effort invested in finding information, analysis, and long discussions within the framework of a team of experts from various fields and are therefore based on broad individual and team knowledge about the future development of an athlete’s sports results. Others are again quick and sudden and prompted by various situations that occur in training and competition settings and require quick reactions from coaches, both in the form of instructions to athletes and in the form of physical interventions that prevent acute injuries to athletes. The developed conceptual framework tries to cover the most comprehensive range of situations that can arise in the training process and possible ways of dealing with them, which should result in different types of decisions and characteristics of coaches’ DM behaviors. For a better understanding of these DM processes of coaches, the conceptual framework uses various sport-specific and general theories related to DM theory and cognitive functioning in these processes.

Figure 2 shows the process perspective of the comprehensive idea of the developed conceptual framework. From this perspective, we can see that all three types of decisions within the training process are interconnected but that there is also a temporal sequence between them, which, in a way, also determines their hierarchy. From this point of view, it is the strategic-type decisions that, at the most general level, direct the training process over a longer period, and its implementation is determined by tactical-type decisions, while the operational-type decisions solve sudden emergency situations and problems that arise when implementing the tactical-type decisions in the time frame of everyday training or competition. An important segment of the process model (Figure 2) is also controlling, which takes place both in training conditions and especially in competitions, which are usually the main milestones for checking the level of implementation of strategic and tactical decisions and represent the accumulated value of the success of the training process. The information obtained in the process of controlling significantly co-shapes the tactical-type decisions for the next period and can also influence changes in the strategic-type decisions. Figure 2 also presents some basic documents and information that represent the input to the process of creating a strategic type of decision, which, in this way, forms the most rational framework for forming this type of decision.

The developed conceptual framework, therefore, foresees three types of decisions, which should have a (1) different role in the comprehensive process of sports training, should be (2) carried out on the basis of different cognitive processes, (3) manifested in the forms of different DM behavior, and (4) should be enforced by using different leadership styles. From this perspective, therefore, each type of decision identified has its own unique role, significance, and meaning. When coaches deal with strategic types of decisions, they have to make decisions that will most comprehensively answer the question, “What do we want to achieve in the selected period of time in order to be successful?” while tactical-type decisions are mostly related to the question, “What do we have to do or how will we implement strategic-type decisions most effectively?.” The operational-type decisions occur suddenly when coaches have to react quickly and make a decision that will adequately solve the emergency (time-constrained) situation and answer the question, “How will we react when something goes wrong, when an error occurs, or when the situation is not according to the plans?”.

For the most valid, reliable, and relevant answers to the questions posed in this way, we need coaches and other experts with different knowledge, experience, and DM behavior to make different types of decisions. This enables the developed conceptual framework to be practically applied in human resources selection processes for DM team formation and the selection of individual coaches for the development and implementation of individual-specific types of decisions. The conceptual framework enables us to understand the (1) specific profile of the most suitable expert for the design and adoption of a particular type of decision from the point of view of the necessary experience, knowledge, and structure of DM styles of an expert, as well as the (2) cognitive processes and risks that are behind individual DM behavior. Therefore, if we are aware of the level of knowledge and the amount of experience of the coaches and/or other experts and we find out (measure) their DM style structure, the conceptual framework gives us relatively precise instructions as to which of the possible candidates is the most suitable for inclusion in the team for designing individual types of decisions or to which coach to entrust the management of athletes in the processes of training and competitions. For instance, if there is an intention to invent an Olympic cycle athlete training plan or national plan for the development of a sports discipline and therefore create strategic-type decisions, chosen candidates for the project team should have a primary (1) extensive knowledge of sports coaching and other important bordered professional disciplines (biomechanics, psychology, physical preparation, nutrition, and so on), (2) they should score high on a rational DM style scale and also have a relatively highly expressed dependent DM style. The team of experts should be led by a highly experienced coach with proven knowledge and the capability to manage the team in a participative/democratic leadership style. In case of formulating tactical-type decisions, a team should be created mainly with highly experienced coaches who can use (1) acquired theoretical knowledge, (2) extensive experience in training and competitions, (3) good knowledge about the functioning of competitional systems, (4) skills in the principles of technical preparations of athletes, and (5) high levels of rational and/or intuitive DM style supported by a dependent DM style and, at the same time, low scores on the avoidant DM style. However, when we search for individuals who can make the most efficient operational-type decisions, we look for coaches or coaches who will operationally run the training processes and, therefore, implement strategical and tactical-type decisions. Therefore, if the coach wants to make valid and effective operational-type decisions (RPD model), the essential conditions are (1) an extensive or sufficient level of domain-specific experiences based on a (2) good theoretical background (knowledge), (3) demonstrated rationality, (4) high scores of intuitive and spontaneous DM style, and (5) a very low level or total absence of expressivity of the avoidant DM style. He needs to be rational in the overall implementation of tactical-type decisions and can react fast and adequately in emergencies.

Regardless of conceptual framework explanations and understandings, there is still an open question of determining the sufficient level of competencies for choosing the experts for the described specific tasks. Since the sufficient or required level and domain of knowledge can be proven and qualified with a degree of formal education and informal theoretical training, the structure of DM styles can be measured, and based on it, given the concerns outlined in the developed conceptual framework, we can draw relatively relevant conclusions about the DM behavior of coaches. However, how do we determine the level of required experience? We need to answer at least two questions about expertise and expert performance in searching for a solution for this inquiry. The first is (1) “What kind of experiences enable coaches to achieve expertise and expert performance?” and second (2) “How long does it take to acquire the expertise of a coach so that he can be considered an expert?.” Both answers can be found in numerous studies conducted by Ericsson and his colleagues over approximately 40 years. They claim that expertise and expert performance can be reached by gaining experiences in the specific-domain environment (1st question) (Ericsson, 2018b, p. 746) based on a long-time involvement (10 years or 10,000 h) in deliberate practice in that domain (2nd question) (Ericsson et al., 1993, p. 372). Although Ericsson (2018b, p. 745) contends that extensive experience in the domain does not invariably lead to an expert level of achievement because some types of experience, such as merely executing the behavior proficiently during routine work without the intention to improve, may not lead to future improvements. Consequently, additional parameters that are outside the experience time frame of involvement in specific-domain high-level training must be determined to determine the expertise and expert performance of the coaches. So, as we wrote at the beginning of that article, athletes’ success can only be measured by the result achieved in the biggest international competitions; the criteria for defining expert coaches in sports can and have to follow the same criteria. Taking that into consideration, the expert coach is different from a novice or non-expert coach in (1) several years of experience in (2) a specific domain of high-level sports training and (3) the constant top results of his athletes at the highest level of international competitions. A coach who meets all three criteria simultaneously can be recognized as an expert, and his decisions, whether long-term, tactical, or sudden, will have relatively high validity and reliability.

However, can novices also make intuitive and spontaneous decisions? Kahneman et al. (2021, p. 54) claim that they can. They probably will if they have a highly expressed spontaneous and/or intuitive DM style in their DM style’s structure. Sudden, spontaneous (also intuitive) prime or initial decisions (solutions) arise quickly after coaches notice the unusual situation. They are consciously aware of prime solutions but not of a process and the use of information (tacit knowledge) to create them (Simon, 1992, str. 155). Thompson et al. (2011, p. 107) assume that the initial, intuitive perception is accompanied by a metacognitive experience, which they call the feeling of rightness of the decision. According to this theory, the System 1 decision (intuitive and spontaneous) develops two responses: (1) the decision and (2) the feeling of rightness of the decision. The feeling of rightness of the decision represents the level of self-confidence about the correctness of the initial (prime) decision and serves as a confirmation of the correctness of the initial (intuitive) solution. Kahneman et al. (2021, p. 54) described judgment as a measurement in which the instrument is a human mind. They also argue that judgment should not be considered the same as a decision and define it as an action that precedes and follows a judgment. They suggest that the feeling of the right judgment is an internal signal of judgment completion, unrelated to any outside information, and that the answer felt right if it seemed to fit comfortably enough with the evidence. They go on to explain that an essential feature of this internal signal is that a sense of coherence is part of the judgmental experience, and it does not depend on the real result and is not contingent on a real outcome. Consequently, an internal signal is just as available for unverifiable judgments (novices) as for true, verifiable (experts) ones.

Furthermore, the strong sense of correctness or familiarity—described by Miller and Ireland (1999, p. 22) as a fundamental characteristic of intuitive judgment (System 1)—is essentially an internal signal indicating judgment completion. It serves as a self-awarded reward for decision-makers upon reaching a conclusion based on their judgment. This is experienced as a satisfying emotional state, a pleasing sense of coherence, where the evidence considered and the resulting judgment feel intuitively “right” (Kahneman et al., 2021, p. 54). Kahneman et al. (2021, p. 166) explain that this internal signal is significant and potentially misleading because it is perceived not as a subjective feeling but as a belief. This emotional experience—where the evidence feels right—disguises itself as rational confidence in the validity of one’s judgment (“I know, even if I do not know why”).

Given this dynamic, delegating decision-making authority to a novice or a coach with insufficient specific experience in scenarios where emergencies are probable and operational-type decisions are required would be highly irresponsible. Their lack of expertise could compromise the quality and reliability of their judgments, potentially leading to ineffective or even harmful outcomes.

## 6 Conclusion

In the present article, the authors developed a conceptual framework for the DM of coaches in sports, which, according to the criterion of structural complexity of movements, are classified as conventional. With the developed conceptual framework, we support the PJDM agenda and upgrade it with theories and concepts that enable direct measurement of the DM behavior of coaches and making conclusions about their individual preferences and ways of acting in different decision contexts, as well as determining the DM styles and structures of the development teams and collective DM bodies when designing future development of sport or an individual athlete. The practical value of the presented model lies primarily in the possibility of using it in selecting relevant experts for various DM situations that arise in the sports training process and, through this, also the detection of risks that may arise due to a wrong choice. Further work in connection with the conceptual framework should aim to empirically verify its interpretable power by using measurements of coaches’ DM styles and establishing their connection with experience, performance, and other demographic characteristics. It would also be expedient to empirically verify the connection and influence of DM styles of coaches and other experts on the success of formed strategic, tactical, and operational-type decisions and on the achieved results of athletes. Our work in preparing this model was largely focused on the characteristics of training and management of athletes in conventional sports (gymnastics), where the foundation of all types of decisions is connected to the greatest extent with the athlete’s technical preparation, which defines his future performance. However, the conceptual framework with appropriate adaptations can also be used to determine the DM behavior of coaches in other sports of monostructural cyclic and polystructural acyclic character.

## Data Availability

The original contributions presented in the study are included in the article/supplementary material, further inquiries can be directed to the corresponding author.

## References

[ref1] Abraham A. Collins D. (2011). Taking the next step: ways forward for coaching science. Quest 63, 366–384. doi: 10.1080/00336297.2011.10483687

[ref2] Abraham A. Collins D. (2015). “Professional judgement and decision making in sport coaching: to jump or not to jump” in International conference on naturalistic decision making 2015 (McLean, VA).

[ref3] Abraham A. Collins D. Martindale R. (2006). The coaching schematic: validation through expert coach consensus. J. Sports Sci. 24, 549–564. doi: 10.1080/02640410500189173, PMID: 16611568

[ref4] Alacreu-Crespo A. Fuentes M. C. Abad-Tortosa D. Cano-Lopez I. Gonzalez E. Serrano M. A. (2019). Spanish validation of general decision-making style scale: sex invariance, sex differences and relationship with personality and coping style. Judgm. Decis. Mak. 14, 739–751. doi: 10.1017/S1930297500005453

[ref5] Al-Hashimi K. Weerakkody V. Elbanna S. Schwarz G. (2021). Strategic decision making and implementation in public organizations in the Gulf cooperation council: the role of procedural rationality. Public Adm. Rev. 82, 905–919. doi: 10.1111/puar.13447, PMID: 39721605

[ref9001] Alkaraan F. Northcott D. (2013). Strategic investment decision-making process: the influence of contextual factors. Meditari Acc. Res. 21, 117–143. doi: 10.1108/MEDAR-09-2012-0031

[ref6] Allwood C. M. Salo I. (2012). Decision-making styles and stress. Int. J. Stress. Manag. 19, 34–47. doi: 10.1037/a0027420

[ref7] Andrew D. P. S. (2004). Effect of congruence of leadership Behaviours on motivation, commitment, and satisfaction of college tennis players, in Department of Sport Management, recreation management, and physical education. Florida, USA: Florida State University: FSU Digital Library.

[ref8] Andrew D. P. S. Kent A. (2007). The impact of perceived leadership Behaviours on satisfaction, commitment, and motivation: an expansion of the multidimensional model of leadership. Int. J. Coach. Sci. 1, 37–58.

[ref9] Ashford M. Abraham A. Poolton J. (2020). A communal language for decision-making in team invasion sports. Int. Sport Coach. J. 8, 122–129. doi: 10.1123/iscj.2019-0062

[ref10] Avsec A. (2012). Do emotionally intelligent individuals use more adaptive decision-making styles? Stud. Psychol. 54, 209–219.

[ref11] Bavoľár J. Orosová O. (2015). Decision-making styles and their associations with decision-making competencies and mental health. Judgm. Decis. Mak. 10, 115–122. doi: 10.1017/S1930297500003223

[ref12] Bayo P. L. Akintokunbo O. O. (2022). Strategic decision making: process and aid to better decision making in organizations: a literature review approach. Int. J. Econ. Bus. Manag. 8, 56–62.

[ref13] Bayram N. Aydemir M. (2017). Decision-making styles and personality traits. Int. J. Recent Adv. Organ. Behav. Decis. Sci. 3, 905–915.

[ref14] Berisha G. Pula J. S. Krasniqi B. (2018). Convergent validity of two decision making style measures. J. Dyn. Decis. Mak. 4, 1–8. doi: 10.2478/jesr-2018-0029

[ref15] Bossard C. Kérivel T. Dugény S. Bagot P. Fontaine T. Kermarrec G. (2022). Naturalistic decision-making in sport: how current advances into recognition primed decision model offer insights for future research in sport settings? Front. Psychol. 13:936140. doi: 10.3389/fpsyg.2022.936140, PMID: 35795439 PMC9252097

[ref16] Calabretta G. Gemser G. Wijnberg N. M. (2017). The interplay between intuition and rationality in strategic decision making: a paradox perspective. Organ. Stud. 38, 365–401. doi: 10.1177/0170840616655483

[ref17] Charbonneau D. Barling J. Kelloway E. K. (2001). Transformational leadership and sports performance: the mediating role of intrinsic motivation. J. Appl. Soc. Psychol. 31, 1521–1534. doi: 10.1111/j.1559-1816.2001.tb02686.x

[ref18] Chelladurai P. Arnott M. (1985). Decision styles in coaching: preferences of basketball players. Res. Q. Exerc. Sport 56, 15–24. doi: 10.1080/02701367.1985.10608426

[ref19] Codreanu A. A. (2016). A VUCA action framework for a VUCA environment. Leadership challenges and solutions. J. Defense Resour. Manag. 7, 31–38.

[ref21] Collins D. Collins L. Carson H. J. (2016). “If it feels right, do it”: intuitive decision making in a sample of high-level sport coaches. Front. Psychol. 7:504. doi: 10.3389/fpsyg.2016.00504, PMID: 27148116 PMC4830814

[ref20] Collins L. Collins D. (2016). Professional judgement and decision-making in adventure sports coaching: the role of interaction. J. Sports Sci. 34, 1231–1239. doi: 10.1080/02640414.2015.1105379, PMID: 26514841

[ref22] Côté J. Salmela J. Trudel P. Baria A. Russell S. (1995). The coaching model: a grounded assessment of expert gymnastics coaches' knowledge. J. Sport Exerc. Psychol. 17, 1–17. doi: 10.1123/jsep.17.1.1

[ref23] Coutts A. J. (2017). Challenges in developing evidence-based practice in high-performance sport. Int. J. Sports Physiol. Perform. 12, 717–718. doi: 10.1123/IJSPP.2017-0455, PMID: 28832264

[ref24] Curşeu P. L. Schruijer S. G. L. (2012). Decision style and rationality: an analysis of predictive validity of the general decision-making style inventory. Educ. Psychol. Meas. 72, 1053–1062. doi: 10.1177/0013164412448066

[ref25] Dean J. W. Sharfman M. P. (1996). Does decision process matter? A study of strategic decision-making effectiveness. Acad. Manag. J. 39, 368–392. doi: 10.2307/256784

[ref26] Dewberry C. Juanchich M. Narendran S. (2013). Decision-making competence in everyday life: the roles of general cognitive styles, decision-making styles and personality. Personal. Individ. Differ. 55, 783–788. doi: 10.1016/j.paid.2013.06.012

[ref27] Dhami M. K. Thomson M. E. (2012). On the relevance of cognitive continuum theory and quasirationality for understanding management judgment and decision making. Eur. Manag. J. 30, 316–326. doi: 10.1016/j.emj.2012.02.002

[ref28] Dunn J. (2006). Coaching decision making in rugby. Available at: https://scholar.google.si/scholar?q=Dunn,+J.+(2006).+Coaching+decision+making+in+rugby.andhl=slandas_sdt=0andas_vis=1andoi=scholart.

[ref30] Elbanna S. Child J. (2007). Influences on strategic decision effectiveness: development and test of an integrative model. Strateg. Manag. J. 28, 431–453. doi: 10.1002/smj.597

[ref31] Elderon W. (2020). Coach to coach. Available at: https://acecoach.com/learner-centred-coaching/.

[ref29] El Othman R. El Othman R. Hallit R. Obeid S. Hallit S. (2020). Personality traits, emotional intelligence and decision-making style in Lebanese universities medical students. BMC Psychol. 8:46. doi: 10.1186/s40359-020-00406-4, PMID: 32370782 PMC7201943

[ref32] Epstein S. (1994). Integration of cognitive and the psychodynamic unconscious. Am. Psychol. 49, 709–724. doi: 10.1037/0003-066X.49.8.709, PMID: 8092614

[ref33] Ericsson A. K. (2018a). “An introduction to the second edition of the Cambridge handbook of expertise and expert performance: its development, organization and content” in The Cambridge handbook of expertise and expert performance. eds. Ericsson K. A. Hoffman R. R. Kozbelt A. Williams A. M. (Cambridge: Cambridge University Press), 3–20.

[ref34] Ericsson A. K. (2018b). “The differential influence on experience, Practise and deliberate practice on development of superior individual performance of experts” in The Cambridge handbook of expertise and expert performance. eds. Ericsson K. A. Hoffman R. R. Kozbelt A. Williams A. M. (Cambridge: Cambridge University Press), 745–769.

[ref35] Ericsson A. K. Krampe R. T. Tesch-Romer C. (1993). The role of deliberate practice in the acquisition of expert performance. Psychol. Rev. 100, 363–406. doi: 10.1037/0033-295X.100.3.363

[ref36] Evans J. S. B. T. Stanovich K. E. (2013). Dual-process theories of higher cognition: advancing the debate. Perspect. Psychol. Sci. 8, 223–241. doi: 10.1177/1745691612460685, PMID: 26172965

[ref37] Faletič L. Avsec A. (2013). Stili odločanja kot napovednik psihičnega blagostanja. [decision-making styles as a predictor of psychological well-being]. Anthropos 3-4, 129–149.

[ref38] Feu S. Ibanez S. J. Gozalo M. Lorenzo A. (2010). Decision and planning style of Spanish handball coaches. Open Sports Sci. J. 3, 111–117. doi: 10.2174/1875399X01003010111, PMID: 37514115

[ref39] Fischer S. Soyez K. Gurtner S. (2015). Adapting Scott and Bruce’s general decision-making style inventory to patient decision making in provider choice. Med. Decis. Mak. 35, 525–532. doi: 10.1177/0272989X15575518, PMID: 25810267

[ref40] Fortin-Guichard D. Thibault N. Tétreault É. Trottier C. Grondin S. (2021). Applying the recognition-primed decision model to differentiate players’ role in volleyball. J. Expertise 4, 248–269. doi: 10.1038/s41598-020-74487x

[ref41] Gambetti E. Fabbri M. Bensi L. Tonetti L. (2008). A contribution to the Italian validation of general decision-style inventory. Personal. Individ. Differ. 44, 842–852. doi: 10.1016/j.paid.2007.10.017

[ref9002] Geisler M. Allwood C. M. (2018). Relating decision-making style to social orientation and time jpproach. J. Behav. Decision Making 31, 415–429. doi: 10.1002/bdm.2066 PMC6032938 30008515

[ref42] Gilson L. L. Goldberg C. B. (2015). Editors’ comment: so, what is a conceptual paper? Group Org. Manag. 40, 127–130. doi: 10.1177/1059601115576425

[ref43] Giske R. Benestad B. Haraldstad K. Høigaard R. (2013). Decision-making styles among Norwegian soccer coaches: an analysis of decision-making style in relation to elite and non-elite coaching and level of playing history. Int. J. Sports Sci. Coach. 8, 689–701. doi: 10.1260/1747-9541.8.4.689

[ref44] Gonzalez-Loureiro M. Vlačić B. (2016). International business decisions and manager’s cognitive style: opening up research avenues from cognitive behavioral strategy. Revista Eletrônica Gestão and Sociedade 10, 1501–1522. doi: 10.21171/ges.v10i27.2131

[ref45] Hammond J. S. Keeney R. L. Raiffa H. (2013). The hidden traps in decision making. *HBR's 10 must reads on making smart decisions*. Boston, MA: Harvard Business Review Press, 1–20. 10185432

[ref9003] Hariri H. Monypenny R. Prideaux M. (2014). Leadership styles and decision-making styles in an Indonesian school context. School Leadership Manag. 34, 284–298. doi: 10.1080/13632434.2013.849678

[ref46] Harren V. A. (1979). A model of career decision-making for college students. J. Vocat. Behav. 14, 119–133. doi: 10.1016/0001-8791(79)90065-4

[ref47] Harris B. S. (2005). Coach and athlete burnout: The role of coach's decision-making style. Morgantown, WV: West Virginia University School of Physical Education.

[ref48] Harvey S. Lyle J. Muir B. (2015). Naturalistic decision making in high performance team sport coaching. Int. Sport Coach. J. 2, 152–168. doi: 10.1123/iscj.2014-0118

[ref49] Heller R. Hindle T. (2001). *Veliki poslovni priročnik* [Essential manager manual]. Ljubljana: Založba Mladinska knjiga.

[ref50] Hickson D. J. Miller S. J. Wilson D. C. (2003). Planned or prioritized? Two options in managing strategic decision-making: process perspectives the implementation of strategic decisions. J. Manag. Stud. 40, 1803–1836. doi: 10.1111/1467-6486.00401

[ref51] Hodges N. J. Franks I. M. (2002). Modelling coaching practice: the role of instruction and demonstration. J. Sports Sci. 20, 793–811. doi: 10.1080/026404102320675648, PMID: 12363296

[ref52] Jaakkola E. (2020). Designing conceptual articles: four approaches. AMS Rev. 10, 18–26. doi: 10.1007/s13162-020-00161-0

[ref53] Jawoosh H. N. Alshukri H. A. Kzar M. H. Kizar M. N. Ameer M. A. A. Razak M. R. A. (2022). Analysis of Coaches' leadership style and its impact on Athletes' satisfaction in university football teams. Int. J. Hum. Move. Sports Sci. 10, 1115–1125. doi: 10.13189/saj.2022.100602

[ref54] Jin H. Kim S. Love A. Jin Y. Zhao J. (2022). Effects of leadership style on coach-athlete relationship, athletes’ motivations, and athlete satisfaction. Front. Psychol. 13:1012953. doi: 10.3389/fpsyg.2022.1012953, PMID: 36578680 PMC9791096

[ref55] Jowet S. Chaundy V. (2004). An investigation into the impact of coach leadership and coach-athlete relationship on group cohesion. Group Dyn. Theory Res. Pract. 8, 302–311. doi: 10.1037/1089-2699.8.4.302

[ref56] Kahneman D. (2017). *Razmišljanje, hitro in počasno* [Thinking, fast and slow]. Ljubljana: UMco d.d.

[ref57] Kahneman D. Frederick S. (2002). “Representativeness revisited: attribute substitution in innovative judgment” in Heuristics and biases: The psychology of intuitive judgment. eds. Gilovich T. Griffin D. Kahneman D. (Cambridge: Cambridge University Press), 49–81.

[ref58] Kahneman D. Klein G. (2009). Condition for intuitive expertise: a failure to disagree. Am. Psychol. 64, 515–526. doi: 10.1037/a0016755, PMID: 19739881

[ref59] Kahneman D. Lovallo D. Sibony O. (2019). A structured approach to strategic decisions. MIT Sloan Manag. Rev. 60, 67–73.

[ref60] Kahneman D. Sibony O. Sunstein C. R. (2021). Hrup. Zakaj tako slabo presojamo. [Noise. A Flaw in Human Judgement]. Ljubljana: UMco d.d.

[ref61] Kaya A. (2014). Decision making by coaches and athletes in sport. Procedia. Soc. Behav. Sci. 152, 333–338. doi: 10.1016/j.sbspro.2014.09.205

[ref62] Kermarrec G. Bossard C. (2014). Defensive soccer players’ decision making: a naturalistic study. J. Cogn. Eng. Decis. Mak. 8, 187–199. doi: 10.1177/1555343414527968

[ref63] Kim H. D. Cruz A. B. (2016). The influence of coaches’ leadership styles on athletes’ satisfaction and team cohesion: a Meta-analytic approach. Int. J. Sports Sci. Coach. 11, 900–909. doi: 10.1177/1747954116676117

[ref64] Klein G. (2008). Naturalistic decision making. J. Hum. Fact. Ergonom. Soc. 50, 456–460. doi: 10.1518/001872008x288385 18689053

[ref65] Klein G. (2015). A naturalistic decision-making perspective on studying intuitive decision making. J. Appl. Res. Mem. Cogn. 4, 164–168. doi: 10.1016/j.jarmac.2015.07.001

[ref66] Klein G. Calderwood R. Clinton-Cirocco A. (2010). Rapid decision making on the fire ground: the original study plus a postscript. J. Cogn. Eng. Decis. Mak. 4, 186–209. doi: 10.1518/155534310X12844000801203

[ref67] Kolar E. Kovač M. Piletič S. (2006). “Ravnanje s športniki v konvencionalnih športnih panogah. [management of the athletes in conventional sports]” in Gimnastika za trenerje in pedagoge 2. eds. Kolar E. Piletič S. (Ljubljana: Gimnastična zveza Slovenije), 10–28.

[ref68] Kolar E. Tušak M. (2022). The decision-making style structure of Slovenian sport managers. Annales Kinesiologiae 13, 47–73. doi: 10.35469/ak.2022.365

[ref69] Kozhevnikov M. (2007). Cognitive styles in the context of modern psychology: toward an integrated framework of cognitive style. Psychol. Bull. 133, 464–481. doi: 10.1037/0033-2909.133.3.464, PMID: 17469987

[ref70] Kruglanski A. W. Gigerenzer G. (2011). Intuitive and deliberate judgments are based on common principles. Psychol. Rev. 118, 97–109. doi: 10.1037/a0020762, PMID: 21244188

[ref71] Langley A. Mintzberg H. Pitcher P. Posada E. Saint-Macary J. (1995). Opening up decision making: the view from the black stool. Organ. Sci. 6, 260–279. doi: 10.1287/orsc.6.3.260, PMID: 19642375

[ref72] Le Menn M. Bossard C. Travassos B. Duarte R. Kermarrec G. (2019). Handball goalkeeper intuitive decision-making: a naturalistic case study. J. Hum. Kinet. 70, 297–308. doi: 10.2478/hukin-2019-0042, PMID: 31915498 PMC6942481

[ref73] Leonard N. H. Scholl R. W. Kowalski K. B. (1999). Information processing style and decision making. J. Organ. Behav. 20, 407–420. doi: 10.1002/(SICI)1099-1379(199905)20:3<407::AID-JOB891>3.0.CO;2-3

[ref74] Leykin Y. DeRubeis R. (2010). Decision-making styles and depressive symptomatology: development of the decision style questionnaire. Judgem. Decis. Mak. 5, 506–515. doi: 10.1017/S1930297500001674

[ref75] Loo R. (2000). A psychometric evaluation of the general decision-making style inventory. Personal. Individ. Differ. 29, 895–905. doi: 10.1016/S0191-8869(99)00241-X

[ref76] Lyle J. (1999). “Coaches’ decision making” in The coaching process: Principles and practice for sport. eds. Cross N. Lyle J. (Oxford: Butterworth-Heinemann), 210–232.

[ref77] Lyle J. W. B. Muir B. (2020). “Coaches’ decision making” in The Routledge international Encyclopaedia of sport and exercise psychology (London: Routledge).

[ref78] Macquet A. C. (2009). Recognition within the decision-making process: a case study of expert volleyball players. J. Appl. Sport Psychol. 21, 64–79. doi: 10.1080/10413200802575759

[ref79] Macquet A. C. Fleurance P. (2007). Naturalistic decision-making in expert badminton players. Ergonomics 50, 1433–1450. doi: 10.1080/00140130701393452, PMID: 17654035

[ref80] Macquet A. C. Kragba K. (2015). What makes basketball players continue with the planned play or change it? A case study of the relationships between sense-making and decision-making. Cogn. Technol. Work 17, 345–353. doi: 10.1007/s10111-015-0332-4

[ref81] Marshall M. K. (2006). The critical factors of coaching practice leading to successful coaching outcomes. Available at: https://aura.antioch.edu/etds/676.

[ref82] Martens R. (2012). Successful coaching. Champaign, IL: Human Kinetics.

[ref83] Martindale A. Collins D. (2007). Enhancing the evaluation of effectiveness with professional judgement and decision making. Sport Psychol. 21, 458–474. doi: 10.1123/tsp.21.4.458

[ref84] Martindale A. Collins D. (2013). The development of professional judgement and decision-making expertise in applied sport psychology. Sport Psychol. 27, 390–399. doi: 10.1123/tsp.27.4.390, PMID: 36626690

[ref85] Matveev L. P. (1977). *Osnovi sportivnoj trenirovki*. [Basics of sports training]. Moskva: Fiskultura i sport.

[ref86] Milazzo N. Fournier J. (2015). Effect of individual implicit video-based perceptual training program on high-skilled karatekas’ decision making. Mov. Sport Sci. 88, 13–19. doi: 10.3917/sm.088.0013, PMID: 18052372

[ref87] Miller C. C. (2008). Decisional comprehensiveness and firm performance: towards a more complete understanding. J. Behav. Decis. Mak. 21, 598–620. doi: 10.1002/bdm.607

[ref88] Miller C. C. Ireland R. D. (1999). Intuition in strategic decision making: friend of toe in the fast-paced 21st century. Acad. Manag. Exec. 19, 19–30.

[ref89] Mitchell J. R. Shepherd D. A. Sharfman M. P. (2011). Erratic strategic decisions: when and why managers are inconsistent in strategic decision making. Strateg. Manag. J. 32, 683–704. doi: 10.1002/smj.905

[ref90] Moen F. Høigaard R. Peters D. M. (2014). Performance progress and leadership behaviour. Int. J. Coach. Sci. 8, 69–81.

[ref91] Mosier K. Fischer U. Hoffman R. R. Klein G. (2018). “Expert professional judgments and naturalistic decision making” in The Cambridge handbook of expertise and expert performance. eds. Ericsson K. A. Hoffman R. R. Kozbelt A. Williams A. M. (Cambridge: Cambridge University Press), 453–476.

[ref92] Musso F. Francioni B. (2012). The influence of decision-making characteristics on the international strategic decision-making process: an SME perspective. Soc. Behav. Sci. 58, 279–288. doi: 10.1016/j.sbspro.2012.09.1002, PMID: 39720782

[ref93] Narooi Z. S. Karazee F. (2015). Investigating the relationship among personality traits, decision-making styles, and attitude of life. Mediterr. J. Soc. Sci. 6, 311–317. doi: 10.5901/mjss.2015.v6n6s6p311

[ref94] Nascimento-Júnior J. R. A. Vissoci J. R. N. Codonhato R. Fortes L. S. Oliveira D. V. Oliveira L. P.. (2018). Effect of the coaches’ leadership style perceived by athletes on team cohesion among elite Brazilian futsal players. Cuadernos de Psicología del Deporte 18, 252–267.

[ref95] Nash C. Collins D. (2006). Tacit knowledge in expert coaching: science or art? Quest 58, 465–477. doi: 10.1080/00336297.2006.10491894, PMID: 39723700

[ref96] Noh Y. K. Lee K. Bum C. H. (2018). The relationship between soccer Club coaches’ decision-making style, basic psychological needs, and intention to continue to exercise: based on amateur male soccer Club members in Korea. Soc. Sci. 7:200. doi: 10.3390/socsci7100200, PMID: 39720620

[ref97] Norman G. R. Grierson L. E. M. Sherbino J. Hamstra S. J. Schmidt H. J. Mamede S. (2018). “Expertise in medicine and surgery” in The Cambridge handbook of expertise and expert performance. eds. Ericsson K. A. Hoffman R. R. Kozbelt A. Williams A. M. (Cambridge: Cambridge University Press), 331–356.

[ref98] Nygren T. E. (2000). “Development of a measure of decision-making styles to predict performance in a dynamic J/DM task” in Paper presented at the 41st Psychonomic society meetings (New Orleans, LA).

[ref99] Papadakis V. M. Barwise P. (1998). “What can we tell managers about making strategic decisions?” in Strategic decisions. eds. Papadakis V. M. Barwise P. (London: Kluwer), 267–287.

[ref100] Post G. van Gelder T. (2023). Seven kinds of decisions sports coaches make. Strateg.: J. Phys. Sport Educ. 36, 24–36. doi: 10.1080/08924562.2023.2238297

[ref9004] Reyna C. Ortiz M. V. Revilla R. G. (2014). Exploratory structural equation modelling of general decision-making style inventory. Revista de Psicologiy 23, 33–39. doi: 10.5354/0719-0581.2014.32872

[ref101] Riaz M. N. Riaz M. A. Batool N. (2012). Personality types as predictors of decision-making styles. J. Behav. Sci. 22, 99–114.

[ref103] Richards P. Collins D. Mascarenhas D. R. (2017). Developing team decision-making: a holistic framework integrating both on-field and off-field pedagogical coaching processes. Sports Coach. Rev. 6, 57–75. doi: 10.1080/21640629.2016.1200819

[ref102] Richards P. Collins D. Mascarenhas D. R. D. (2012). Developing rapid high-pressure team decision-making skills. The integration of slow deliberate reflective learning within the competitive performance environment: a case study of elite netball. *International journal of*. Reflective Pract. 13, 407–424. doi: 10.1080/14623943.2012.670111, PMID: 39723700

[ref104] Rowe A. J. Mason O. R. (1987). Managing with style: A guide to understanding, assessing, and improving decision making. San Francisco, CA: Jossey-Bass.

[ref105] Rozman R. Kovač J. (2012). Management. Ljubljana: Založba GV.

[ref106] Satpute A. B. Lieberman M. D. (2006). Integrating automatic and controlled processing into neurocognitive models of social cognition. Brain Res. 1079, 86–97. doi: 10.1016/j.brainres.2006.01.005, PMID: 16490183

[ref107] Schoemaker A. F. (2010). The relationship between decision-making style and negative affect in college students. (Master thesis). Philadelphia: [a.F. Schoemaker]. Available at: https://idea.library.drexel.edu/islandora/object/idea%3A3334.

[ref108] Scott S. G. Bruce R. A. (1995). Decision-making style: the development and assessment of a new measure. Educ. Psychol. Meas. 55, 818–831. doi: 10.1177/0013164495055005017

[ref109] Secchi D. (2010). Extendable rationality. Understanding decision making in organizations. New York: Springer Publishing.

[ref110] Sherman C. A. Fuller R. Speed H. D. (2002). Gender comparison of preferred coaching behaviours in Australian sports. J. Sport Behav. 23, 389–406.

[ref112] Simon H. A. (1979). Rational decision making in business organizations. Am. Econ. Rev. 89, 493–513.

[ref113] Simon H. A. (1987). Making management decision: the role of intuition and emotion. Acad. Manag. Exec. 1, 57–64.

[ref114] Simon H. A. (1992). What is an “explanation” of behaviour? Psychol. Sci. 3, 150–161. doi: 10.1111/j.1467-9280.1992.tb00017.x

[ref111] Simon H. A. (1976). “From substantive to procedural rationality” in Method and appraisal in economics. ed. Latsis V. S. J. (New York: Cambridge University Press), 129–148.

[ref115] Spicer D. P. Sadler-Smith E. (2005). An examination of the general decision-making style questionnaire in two UK samples. J. Manag. Psychol. 20, 137–149. doi: 10.1108/02683940510579777

[ref116] Stanovich K. E. (1999). Who is rational? Studies of individual differences in reasoning. Mahwah, NY: Erlbaum.

[ref117] Taggart W. Valenzi E. (1990). Assessing rational and intuitive style: a human information processing metaphor. J. Manag. Stud. 27, 149–172. doi: 10.1111/j.1467-6486.1990.tb00758.x

[ref118] Thompson V. A. Prowse Turner J. P. Pennycock G. (2011). Intuition, reason and metacognition. Cogn. Psychol. 63, 107–140. doi: 10.1016/j.cogpsych.2011.06.001, PMID: 21798215

[ref119] Thunholm P. (2004). Decision-making style: habit, style or both? Personal. Individ. Differ. 36, 931–944. doi: 10.1016/S0191-8869(03)00162-4

[ref120] Till K. A. Muir R. Abraham A. Piggott D. Tee J. (2019). A framework for decision-making within strength and conditioning coaching. Strength Condit. J. 41, 14–26. doi: 10.1519/SSC.0000000000000408

[ref121] Tomić M. (2007). Sportski menadžment. [Management in sport]. Beograd: Fakultet sporta i fizičkog vaspitanja.

[ref122] Tversky A. Kahneman D. (1974). Judgment under uncertainty: heuristics and bias. Science 185, 1124–1131. doi: 10.1126/science.185.4157.1124, PMID: 17835457

[ref123] Tversky A. Kahneman D. (1981). The framing of decisions and the psychology of choice. Science 211, 453–458. doi: 10.1126/science.7455683, PMID: 7455683

[ref124] Voight M. (2002). Improving the quality of training, coach and player responsibilities. J. Phys. Educ. Recreat. Dance 73, 43–48. doi: 10.1080/07303084.2002.10607828

[ref125] Wilson P. J. Kiely J. (2023). Developing decision-making expertise in professional sports staff: what we can learn from the good judgement project. Sports Med. Open 9, 100–109. doi: 10.1186/s40798-023-00629-w, PMID: 37878189 PMC10600061

[ref126] Wu A. M. Lai M. H. Chan I. T. (2014). Coaching behaviours, satisfaction of needs, and intrinsic motivation among Chinese university athletes. J. Appl. Sport Psychol. 26, 334–348. doi: 10.1080/10413200.2014.888107

